# Balancing patients’ fears of recurrence and fears of COVID-19 when considering their preference for review consultations

**DOI:** 10.1007/s00405-021-06662-3

**Published:** 2021-02-13

**Authors:** Joanna Dimelow, Derek Lowe, Simon N. Rogers

**Affiliations:** 1 Liverpool Head and Neck Centre, Liverpool University Hospital, Lower Lane, Liverpool, L9 1AE UK; 2 Astraglobe Ltd, Congleton, Cheshire UK; 3 Faculty of Health and Social Care and Medicine, Edge Hill University, St Helens Road, Ormskirk, UK

**Keywords:** COVID-19, Head and neck cancer, Fear of recurrence, Follow-up consultations

## Abstract

**Purpose:**

Head and neck cancer (HNC) patients may experience fears regarding cancer recurrence (FoR) and of catching COVID-19. There could be unease for attending hospital clinics for face to face (F2F) examination. F2F benefit in cancer surveillance has to be balanced against the risk of virus transmission. This study aimed to report perceptions of fear of cancer and fear of COVID-19 and to report patient preference for follow-up consultation in HNC survivors during the COVID-19 pandemic.

**Methods:**

The study ran from lockdown in England on 24th March to 29th July 2020. Patients were offered preference to postpone their consultation, to have it by telephone, or F2F. A postal survey was undertaken in the 2 weeks post-consultation (actual or postponed).

**Results:**

There were 103 patients. Initial action by consultant and patient resulted in 51 postponed consultations, 35 telephone consultations and 17 F2F meetings, with 10 F2F triggered by the patient. There were 58 responders to the survey and most (39) had a clear preference for one mode of follow-up consultation during the COVID-19 pandemic, with half (19) preferring F2F. A similar response was seen regarding their consultations in general to address unmet needs and concerns, with 38 having a preferred mode, 29 preferring F2F. Serious fears about recurrence and COVID-19 were at relatively low levels with a tendency to be more concerned about recurrence.

**Conclusion:**

Any redesign of mode and frequency of out-patient follow-up in light of COVID-19 should be undertaken in discussion with patient groups and with individual patients.

## Introduction

There are recommendations concerning the frequency of patient review following treatment of head and neck cancer (HNC) [1–4]. These follow-up consultations traditionally take place face to face in clinic and allow an opportunity to assess treatment response, identify recurrence and manage complications [5]. Fear of recurrence is a key reason why patients attend [6] and assessment is usually performed by palpation and visual inspection, either directly or via endoscopy.

In England on the 24th March 2020, ‘lockdown’ occurred due to the COVID-19 pandemic. There was huge disruption of out-patient services. For a period, all out-patient consultations were stopped [7]. To reduce transmission and infection risks, following ‘lockdown’, there was an imperative for non-face to face review either by telephone or telemedicine [8]. There was substantial fear of COVID-19 amongst the population including patients, their careers, and staff [9]. This psychological threat led to the development of various fear of COVID-19 questionnaires [10, 11]. For HNC patients, their fear of COVID-19 had to be balanced against their perceived risk of recurrence. Remote review has limitations because it lacks the physical check and patients might feel that a recurrence could be missed without a physical examination and that a remote consultation was less reassuring in this regard.

As there is the potential for unease between the patients view of recurrence and the benefit of face to face checks compared to their fear of the COVID when venturing out to the hospital clinic, the aim of this study was to report perceptions of fear of cancer and fear of COVID and to report patient preference for follow-up consultation in head and neck survivors during the first months of the COVID-19 pandemic. The hypothesis is that because of the fear of COVID-19 patients will trade off their fear of recurrence and opt for a postponement of their scheduled review or a non-face to face consultation. Our hope is that, by sharing the patients perspective this might inform not only current out-patient review strategies but also any changes that might occur to stratified follow-up when the pandemic has receded or is over.

## Methods

Following lockdown in England on 24th March 2020, the hospital sent all patients expecting review consultations/clinic appointments a standard letter informing them that their clinic had been postponed. This study took place from then up to 29th July 2020. A consecutive series of previously treated head and neck cancer patients were eligible. New referrals, non-cancer, and palliative patients were not included.

A three step approach was proposed to manage existing patients. First, a consultant review of the clinic list was made a couple of weeks in advance of the next scheduled appointment, with patients allocated into groups, either a postponed appointment, telephone consultation or a face to face consultation. Allocation was based on the potential risk of recurrence and influenced by factors such as time since treatment, tumour stage, resection margins, and time since last face to face consultation. The consultant’s secretary would then phone those whose consultations could be postponed to ascertain if they were accepting of the allocation or if they had a different preference. The consultant was informed if a patient had a problem and then either a telephone consultation was made or a clinic review arranged. A record of all postponed patients was made to make sure they remained under follow-up. Second, those allocated for telephone consultation review were sent a Patient Concerns Inventory (PCI-HN) prompt list [8] a week beforehand together with the invitation about the expected call. The call would take place in a morning or afternoon slot as allocated, without stating a precise time to allow some flexibility. As usual a letter was written as part of the post-consultation. If needed, for patients with problems, a face to face consultation was arranged. Third, a face to face consultation was arranged for patients calling with an urgent problem, patients following the telephone consultation who needed review, and also those who had appointments postponed and needed to be checked out. However, there were some study patients who actually took it upon themselves to ring the unit to get an appointment (telephone or face to face) and were not allocated into groups by the consultant.

**Fig. 1:**
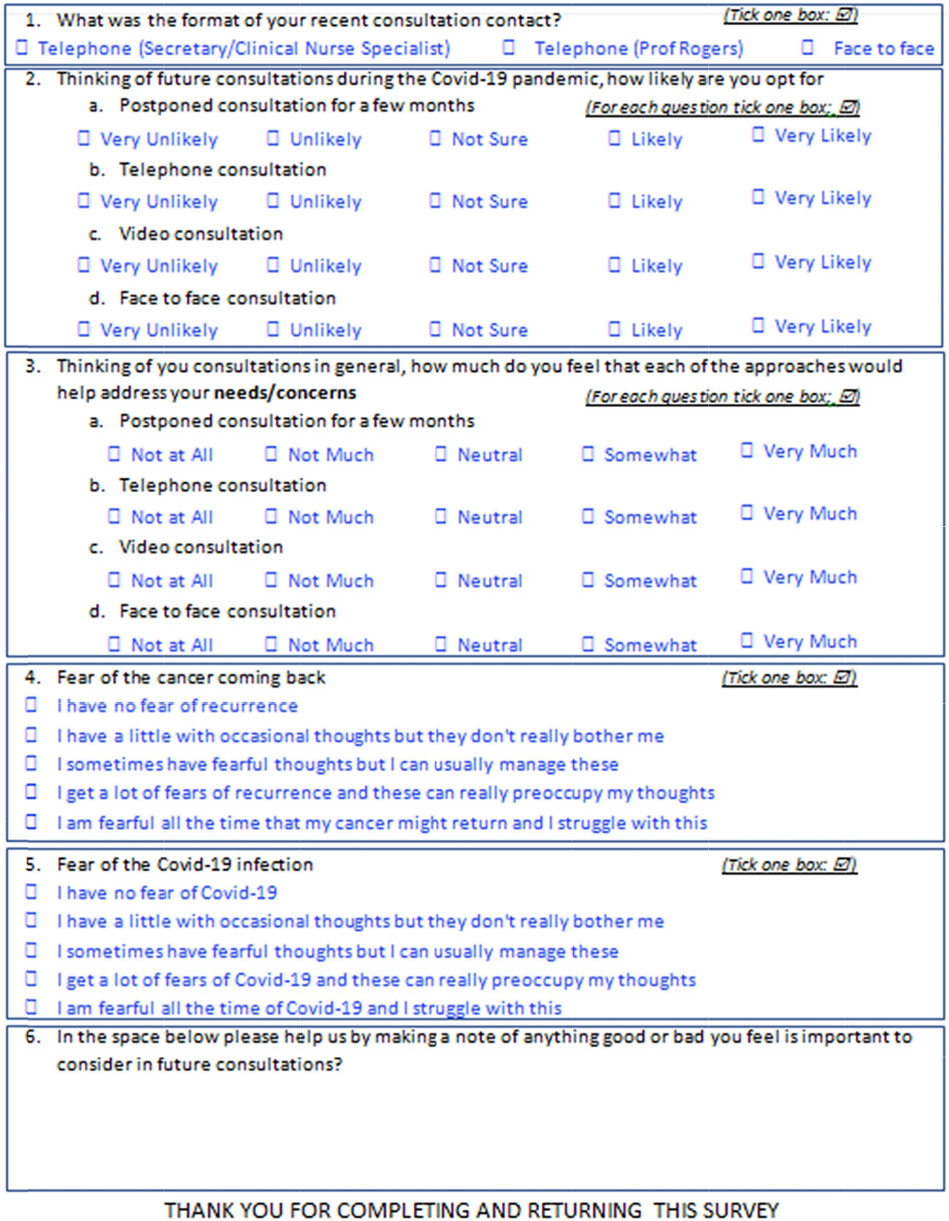
Patient questionnaire

In the 2 weeks after having a first contact with the consultant (telephone or face to face), or in the 2 weeks after the postponement, patients were sent a short questionnaire (Fig. 1) to complete and return. This questionnaire included a question previously developed for measuring fears of recurrence [12] and this was modified for this study into a question for measuring fears of COVID-19. No reminders were sent. Patients were informed in the accompanying letter that through this questionnaire the Trust hoped to gain an understanding of what method of consultation their patients preferred to inform the provision of their service in future. Clinical information retrieved from the hospital patient record system was anonymised and categorised as: age (< 55, 55–64, 65–74,75 or over), gender (male, female), clinical stage (early T1N0/T2N0, or late), site (oral, oropharyngeal, laryngeal, other), osteoradionecrosis (Y/N), surgery (Y/N), free flap (non, soft, composite), radiotherapy (Y/N), chemotherapy (Y/N) and time since primary diagnosis (< 12 months, 12–23, 24–59, 60 months or more).

Fisher’s exact test was used to compare response rates between patient subgroups and the Spearman correlation coefficient (*r*_*s*_) was used to assess the amount of correlation between fears of COVID-19 and fears of cancer returning, and the Kappa coefficient was used to estimate the strength of agreement.

## Results

Clinical characteristics of the 103 study patients are shown in Table 1. The initial action from the consultant was to postpone the appointments of 51 patients, to have a telephone consultation with 25, to have a face to face contact with 6 patients, and 21 rang in to make their own arrangements. Any changes to the initial actions are shown in Table 2. The outcome of all this was that scheduled appointments were postponed for 51, became telephone consultations for 35 and remained face to face for 17. For the 52 patients within 5 years of primary diagnosis 22 were postponed, 19 were by telephone and 11 were face to face; for the 51 patients beyond 5 years in follow-up 29 were postponed, 16 were by telephone and 6 were face to face. Most (10) of the 17 face to face consultations were from patients who rang in to ask for them. The questionnaire had 58 responders (56%) with no obvious response biases by clinical characteristics (Table 1) nor by initial action taken by the consultant or the subsequent agreed type of consultation (Table 2).

**Table 1:** Casemix of the 103 study patients and response to the study questionnaire

	Patients	Questionnaire response		*P* value*
		%	*N*	
ALL patients	103	56	58	
Gender				
Male	64	59	38	0.54
Female	39	51	20	
Age				
< 55	16	44	7	0.71
55–64	26	62	16	
65–74	25	60	15	
≥ 75	36	56	20	
Clinical stage				
Early (T1N0 or T2N0)	42	62	26	0.42
Late	61	52	32	
Tumour site				
Oral	58	55	32	0.48
Oropharynx	29	62	18	
Larynx	2	100	2	
Other	14	43	6	
Treatment				
Surgery only	33	55	18	0.30
Radiotherapy only	10	80	8	
Radiotherapy and chemotherapy	13	46	6	
Surgery and radiotherapy	41	51	21	
Surgery and radiotherapy and chemotherapy	6	83	5	
Free-flap transfer(surgery only)				
Composite	12	58	7	0.61
Soft	24	63	15	
None	44	50	22	
Review status				
Primary cancer only	56	54	30	
Recurrence/2nd primary	17	59	10	0.83
ORN only	30	60	18	
Months from primary diagnosis				
< 12	12	50	6	0.59
12–23	12	75	9	
24–59	28	54	15	
≥ 60	51	55	28	

^*^Fisher’s exact test

**Table 2:** Summary of the initial actions decided by the consultant and subsequent changes

Initial Action from consultant	Action changed	Actual type of clinic agreed			Total
		No clinic	Telephone	Face2Face	
Postponed	No	42 (21)	–	–	42 (21)
	Yes	–	8 (6)	1 (0)	9 (6)
Telephone	No	–	14 (8)	–	14 (8)
	Yes	9 (7)	–	2 (1)	11 (8)
Face2Face	No	–	–	4 (3)	4 (3)
	Yes	–	2 (2)	–	2 (2)
None: patient rang to get appointment		–	11 (6)	10 (4)	21 (10)
Total		51 (28)	35 (22)	17 (8)	103 (58)

Table shows total number of patients (number responding to the study questionnaire)

When asked to think about future consultations during the COVID-19 pandemic (Table 3) most patients were comfortable with having face to face contact (60% ‘likely’ or ‘very likely’) though nearly half seemed happy to postpone for a few months (43%) or to have a telephone consultation (45%) and but were less drawn towards a video linkage (10%). The strongest level of preference for 40 of the 58 responders was ‘very likely’, 16 ‘likely’ and 2 ‘not sure’. For 39, their strongest preference was for one type only (19 face to face, 10 postpone, 9 telephone, 1 video), while 12 could not decide between two types (4 telephone or face, 4 postpone or face, 2 postpone or telephone, 1 video or face, 1 telephone or video). Seven patients could not decide between three types (3 postpone or telephone or face, 3 postpone or telephone or video, 1 telephone, video or face).

**Table 3:** Questionnaire responses in regard to suitability of the type of consultation

	Very Unlikely	Unlikely	Not sure	Not stated	Likely	Very Likely	% Likely/V likely
Thinking of future consultations during the COVID-19 pandemic, how likely are you to opt for:							
Postponed for a few months	12	11	4	6	13	12	43
Telephone	9	11	4	8	11	15	45
Video	21	10	9	12	4	2	10
Face 2 face	2	3	8	10	12	23	60

	Not at all	Not much	Neutral	Not stated	Somewhat	Very Much	% Somewhat /V much
Thinking of your consultations in general, how much do you feel that each of the approaches would help address your needs/concerns:							
Postponed for a few months	11	9	18	5	8	7	26
Telephone	12	12	9	8	5	12	29
Video	25	6	8	10	6	3	16
Face 2 face	2	2	7	7	9	31	69

When asked to think about their consultations in general and how well each approach would help address their needs/concerns (Table 3) more patients felt that face to face provided this (69% ‘somewhat’ or ‘very much’) than the other options (29% telephone, 26% postpone, 16% video). The strongest level of preference for 41 of the 58 was ‘very much’, 10 ‘somewhat’, 6 ‘neutral’ and 1 ‘not much’. For 38, their strongest feeling was for one type only (29 face to face, 4 postpone, 4 telephone, 1 video), while 13 could not decide between two types (3 telephone or face, 3 postpone or face, 2 postpone or telephone, 2 video or face, 2 telephone or video, 1 postpone or video). Four patients could not decide between three types (postpone or telephone or face) and 3 could not decide between all four types (1 ‘somewhat’ for all, 1 ‘neutral’ for all, 1 ‘not much’ for all).

Fears of getting the COVID-19 virus were slightly lower than fears of cancer recurrence (Table 4) in that 34 had little or no fears of the virus compared to 21 of having recurrence. Similar numbers (7 virus, 9 recurrence) either had a lot of fears or were fearful all the time, with 4 patients having significant fears for both and 12 patients with either or both. The actual type of clinic initially agreed with these 12 patients was to postpone for 6, to hold a telephone consultation for 4 and to meet face to face for 2. Spearman correlation between the two types of fear was *r*_*s*_ = 0.54, *p* < 0.001. The kappa coefficient of 0.27 indicated a less than moderate level of agreement; 28 patients had the same level of fear for both, 23 feared recurrence more than COVID-19 and only 7 feared COVID-19 over recurrence. For 30 patients within 5 years of primary diagnosis, 17 had the same level of fear for both, 12 had more fear about recurrence more while 1 had more fear of COVID-19; for 28 patients beyond 5 years in follow-up, 11 had the same level of fear for both, 11 had more fear of recurrence while 6 had more fear about COVID-19.

**Table 4:** Questionnaire responses in regard to fears of recurrence and fears of Covid-19

Fear of recurrence	Fear of Covid-19^a^					Total
	1	2	3	4	5	
1. I have no fear of recurrence	2	1	–	–	–	3
2. I have a little fear with occasional thoughts but they don’t really bother me	4	12	2	–	–	18
3. I sometimes have fearful thoughts but I can usually manage these	2	11	12	3	–	28
4. I get a lot of fears of recurrence and these can really preoccupy my thoughts	–	1	2	1	1	5
5. I am fearful all the time that my cancer might return and I struggle with this	1	–	1	1	1	4
Total	9	25	17	5	2	58

^a^1. I have no fear of COVID-19, 2. I have a little fear with occasional thoughts but they don’t really bother me, 3. I sometimes have fearful thoughts but I can usually manage these, 4. I get a lot of fears of COVID-19 and these can really preoccupy my thoughts, 5. I am fearful all the time of COVID-19 and I struggle with this

## Discussion

Follow-up assessment after completion of HNC treatment is a fundamental aspect of care. Prior to COVID-19, there has been debate about how out-patient consultations could become more individualised and stratified [13]. The pandemic has accelerated the need for change as although a huge unexpected threat, paradoxically, the COVID-19 pandemic has been an opportunity to reflect on how to do things differently. There have been changes to how out-patient clinics are performed. As fears of recurrence are an important element of HNC review consultations perhaps by considering both fears of COVID-19 and fears of recurrence, this might help inform and shape expectations. This novel study gained initial perceptions of a consecutive group of HNC patients following lockdown. The response rate was reasonable without any obvious responder bias by clinical characteristics. Unfortunately, audit approval did not allow for a reminder survey to be sent to non-responders. The findings are limited to the practice of one consultant in one hospital and although there was a range of patients by clinical characteristics, most were oral cancer reviews. Nearly half of the group were longer than 5 years in follow-up and this reflected the number of patients followed up for osteoradionecrosis. The findings of this study might have been different in different cancer sites (oropharynx, larynx) and in patients closer to treatment. In this study, it was not possible to include one of the emerging fear of COVID-19 scales [10, 11]. The study reports the early experience of patients post lockdown and does not assess their long-term anxieties around COVID-19 and out-patient review preferences. This study does not include the views amongst healthcare professionals, who will have varying degree of COVID-19-related anxieties in terms of HNC where examination of the mouth and throat and the use of aerosol producing procedure such as nasendoscopy carry additional risk. Finally, it was not the intention to validate the selection criteria on favouring the type of review. However, there is merit to develop decision process tools to help patients and clinicians weigh up the need and benefit of having a consultation, whether face to face or virtual, or alternatively being discharged back to primary care. In this study no specific objective criteria were used to decide the initial allocation and it was a combination of factors that might suggest that recurrence was more likely and might be missed without physical examination. Thus, with this subjective approach, it is not possible to give sufficient level of detail or propose an algorithm that would allow other clinicians to make the same decision.

Given the context of the COVID-19 lockdown, it is perhaps of little surprise that the initial tendency for both the patient and consultant was to postpone the appointments. Most of the appointments that took place were by telephone while most of those face to face were requested from patients themselves. The inference is that patients sought face to face if they perceived a problem. In addition, one fifth of patients on review contacted the clinician’s secretary to seek advice about their rescheduling consultation, or bring forward. This strategy would significantly reduce follow-ups but has the risk of missing asymptomatic evidence of recurrence and possibly earlier detection of recurrence. Previous reports have brought into question the reliability of patients to bring forward their scheduled appointment [14], but any reticence might be overcome if the patients have clear instruction regarding signs and symptoms of possible recurrence and a straightforward and reliable means of getting in touch. Although further evaluation is necessary, telephone consultations could be a very convenient way to have a conversation around the patients’ progress and address any unmet needs. A prompt list sent out to the patient in advance of the consultation (Patient Concerns Inventory) could be a useful adjunct [8]. The patients were much less sure about video linked telemedicine approaches. The COVID-19 pandemic is likely to accelerate the wider use of this approach but further evaluation is needed in terms of patient preference and clinical benefit.

In this study, it seems that patients were less fearful of COVID-19 than recurrence. It is very reasonable and natural for patients to have fears and only a small number had substantial fears. Some patients were undecided regarding a preference between telephone or F2F as the mode for future consultations. This probably reflects uncertainly in regard to their future symptoms and the guidance regarding virus risk during the pandemic. Their preference might strengthen as the perceived threat of the virus diminishes. It would be worthwhile repeating the study as the potential treat of COVID might increase or diminish depending on the behaviour of the pandemic. This would impact on patients fears related to attending consultations. However, the premise of the balance of risk, as touched on in this paper, is pertinent to the first wave and it is possible to tentatively extrapolate to future concerns and raise the issue of patient preference around the type of consultation. Clinicians recognise that COVID-19 has driven a change to more virtual clinics [15]; however, it remains unclear as to how practice over the long-term will change as services return to some sense of normalcy. The future preference of patients, as the pandemic wanes is uncertain, though face to face seems to be favoured compared to other methods. Another area of uncertainty is the frequency of contact and the use of scans to check for further HNC.

## Conclusion

A better understanding of review consultation patient preference together with the increased use of telephone and video consultations has potential to reduce the number of face to face appointments. This would free up capacity in the clinic setting and this is important given the issue of social distancing and time taken to clean the clinical environment and equipment between patients. Ongoing evaluation is needed and it would be beneficial to keep asking patients for their preference, continue to record type and frequency of review, and assess the clinical presentation of treatment failures and determine if change in out-patient follow-up strategy compromised this.
